# Organic fluorescent nanoprobes with NIR‐IIb characteristics for deep learning

**DOI:** 10.1002/EXP.20210097

**Published:** 2022-02-19

**Authors:** Kevin Wanderi, Zongqiang Cui

**Affiliations:** ^1^ State Key Laboratory of Virology Wuhan Institute of Virology Center for Biosafety Mega‐Science Chinese Academy of Sciences Wuhan China; ^2^ Department of Analytical Microbiology and Nanobiology University of Chinese Academy of Sciences Beijing China

**Keywords:** deep learning, nanotechnology, NIR‐IIb window, organic fluorescent nanoprobes, small‐animals imaging

## Abstract

Illumination of biological events with near‐infrared II sub‐channel (NIR‐IIb, 1500–1700 nm) enhances the transparency of biological tissues, which is very attractive for deep imaging. Due to the long‐wavelength, which reduces optical damage, suppresses autofluorescence, and obviates light scattering, NIR‐IIb nanoprobes afford deep tissue penetration with unprecedented spatiotemporal resolution. Hence, NIR‐IIb imaging facilitates deep learning and decipherment of biological proceedings in living organisms with astounding high clarity. In comparison to its predecessors in the visible‐near‐infrared spectrum, imaging in the NIR‐IIb has shown great potential for tissue imaging and extrapolating imaging applications for clinical studies. However, the use of organic fluorescent nanoprobes (OFNPs) in the NIR‐IIb region is still rare since it is in its early stages. Thus, herein we aim to survey the recent development of different organic fluorescent nanomaterials with NIR‐IIb characteristics, their unique photophysical properties, and their utilization in deep imaging in animal models. Further, practical researches on organic fluorescent nanoprobes with NIR‐IIb emission and their transition to clinical applications are highlighted.

## Graphical abstract

This paper reviews the recent progresses of organic fluorescent nanoprobes (OFNPs) with NIR‐IIb emission, focusing on their construction strategies, optical properties, and their applications in deep learning in small animal models such as cerebrovascular imaging, tumor imaging, intestinal imaging, and systemic vascular imaging. In addition, the challenges and potential future research based on NIR‐IIb fluorescent nanoprobes are highlighted.

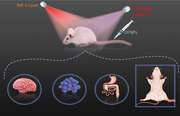

## 1 INTRODUCTION

Up to date, obtaining details of biological events in a wholesome noninvasive approach in living organisms is still a great challenge.^[^ ^1^ ^]^ Even though over the years, scientists have made substantial progress to enable whole‐body imaging by unraveling the thorny resolution/penetration conundrum using tomographic imaging modalities like computed tomography (CT), Ultrasound imaging (US), Single‐photon emission CT (SPECT), magnetic resonance imaging (MRI), and Positron emission tomography (PET).^[^ ^2^ ^]^ For anatomical imaging, CT, US, or MRI are usually preferred at a clinical level. These different techniques have advantages and disadvantages. For example, ultrasound images acquired based on acoustic resistance between tissues boundaries, are characterized by high spatiotemporal resolution, limited depth penetration of between 10 and 150 mm, and low contrast.^[^ ^3^ ^]^ As for X‐ray CT, images are based on X‐ray reduction by different tissues and are characterized with excellent spatial resolution, unlimited depth penetration of >500 mm, and low sensitivity for soft tissues. Thus, the technique is preferentially used for imaging bones.^[^ ^4^ ^]^ For MRI, images are acquired using emitted radio frequency signals and strong magnetic fields which affords unlimited depth penetration >500 mm, high spatial resolution, and low sensitivity.^[^ ^5^ ^]^ Although this model has a lower spatial resolution than CT, it is more suitable for soft tissue imaging. For PET and SPECT, images are acquired using radioisotopes‐labeled agents, the difference being the use of single‐photon emitting isotopes in SPECT. In addition, PET is superior to SPECT and more expensive. In terms of advantages both display unlimited depth penetration (>500 mm), high sensitivity(pM‐nM), low spatial resolution (≤2 mm), and minimal anatomical information.^[^ ^6^, ^7^ ^]^ Indeed, many of these technologies have played a crucial part in transforming prognosis and clinical treatment.^[^ ^8^, ^9^, ^10^ ^]^Nevertheless, precision with great sensitivity and maximum spatiotemporal resolution, is still a major hindrance to their application for in vivo imaging. Thus, photoluminescence imaging strategies such as optical fluorescence imaging that offers elevated sensitivity and high spatiotemporal resolution in a non‐invasive wholesome manner at a low cost are preferred.^[^ ^11^ ^]^ Furthermore, it permits the visualization of multiple biological events in living animals due to its large optical window (400–2500 nm).^[^ ^12^, ^13^ ^]^

Since its introduction in studying life processes, fluorescence imaging technology has metamorphosized into a mainstay in basic research and tremendously aided in broadening our apprehension of the intriguing complexity of biological processes that dictate life in living organisms.^[^ ^14^, ^15^, ^16^, ^17^ ^]^ Hence, fluorescence imaging plays an instrumental role in transforming fundamental biological research and biomedical research at the preclinical and clinical levels.^[^ ^18^, ^19^ ^]^ Despite, all its success at a cellular level and its appeal to enable whole‐body imaging, its transition to in vivo visualization has often been impeded by an opaque barrier caused by biological tissues.^[^ ^20^, ^21^ ^]^Tissues have the inclination to absorb, disperse photons, and cause strong autofluorescence. Thus, in vivo imaging with traditional channels often results in a low signal/background ratio and limited biotissue penetration depth.^[^ ^22^ ^]^ This is also exacerbated by the fact that these biological tissues also contain other constituents that absorbs photons in the Near‐infrared region which further obscures the interrogation window of optical fluorescence imaging in both ultra‐violet‐visible and infrared wavelength spectrum.^[^ ^23^ ^]^ Hence, the expansion of novel fluorescent probes and imaging technologies that can non‐invasively overcome biotissues barriers are needed to facilitate the deep learning of biological processes.^[^ ^24^, ^25^, ^26^ ^]^ Hitherto, superior imaging instrumentations such as two‐ and multi‐photon microscopy,^[^ ^27^, ^28^ ^]^ light‐sheet microscopy,^[^ ^29^, ^30^ ^]^ confocal microscopy,^[^ ^31^ ^]^ optical coherence tomography,^[^ ^32^ ^]^ and fluorescence‐mediated tomography^[^ ^33^ ^]^ have been reported to be applied for in vivo imaging in the second near‐infrared window. Contemporaneously, on the other hand, to faithfully increase tissue depth penetration with a high signal/background ratio more emphasis has been shifted to pursue the development of superior fluorescent probes with long‐wavelength in the Near‐infrared‐II region with high photoluminescence quantum yield, high optical stability, and high biocompatibility.^[^ ^34^ ^]^ To a large extent, this has been facilitated by the rapid expansion of nanotechnology over the last three decades.^[^ ^35^ ^]^ Novel fluorescent nanoprobes with NIR‐II characteristics (1000–1700 nm) exhibiting better optical properties such as scanty optical scattering and almost zero autofluorescence are available, enabling unprecedented deep visualization of biological events with super‐resolution.^[^ ^36^ ^]^ In contrast to their traditional counterparts in the UV‐Vis/Infrared spectrum or Near‐infrared I, Near‐infrared II fluorescent nanoprobes have long wavelengths that display impressively high brightness, excellent optical stability, and high absorption coefficients. Furthermore, they are endowed with a large surface area which provides a platform for their functionalization/optimizing targeting, leading to the application of these novel hybrid nanomaterials in bio‐sensing tracking, biomedical research, and bionanotechnology.^[^ ^37^, ^38^ ^]^

Near‐infrared II/shortwave‐infrared window (NIR‐II/SWIR) which is also widely known as the transparent biological window can broadly be classified into two groups, Near‐infrared IIa (1000–1300 nm) and Near‐infrared IIb (1500–1700 nm) window.^[^ ^39^ ^]^ This is due to the vibrational overtone of water at either 1444 or 1944 nm. Thus even though imaging in the Near‐infrared sub‐channel provides the required deep penetration for deep learning, there is also the problem of increased optical attenuation due to the increased water absorption of the region. Nonetheless, the biological tissue attenuation coefficients are much low in the NIR‐IIb (1500–1700 nm) region than in the other regions, leading to deeper biotissue penetration and scanty autofluorescence. This results in a high signal‐to‐noise ratio (SNR) which in turn facilitates deep learning of biological events in the NIR‐IIb spectrum. However, fluorescent probes with NIR‐IIb emission are few and are currently in a developmental stage. In recent years, substantial breakthroughs have resulted in much improved NIR‐IIb fluorescent nanoprobes revamping their utilization in deep learning of the proceedings in biological tissues, in vivo blood flow tailing, whole‐body vasculature imaging, brain vasculature monitoring, tumor recognition, and image‐guided treatment.^[^ ^40^, ^41^ ^]^

**FIGURE 1:**
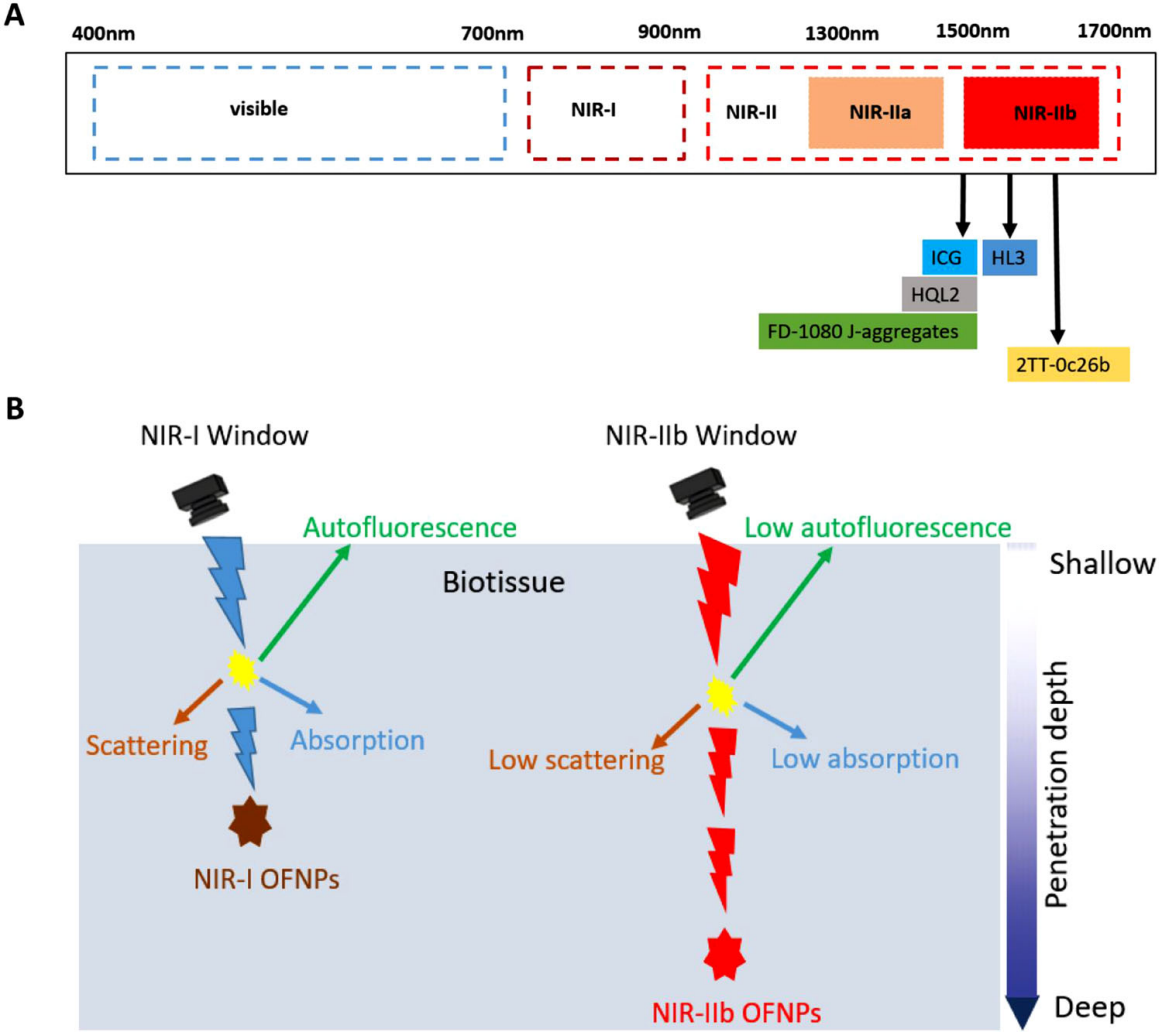
(A) Organic fluorescent nanoprobes (OFNPs) with NIR‐IIb emission positioned by their corresponding maximum tail emission (*λ*_max,em_) along the electromagnetic spectrum windows. (B) Schematic diagram of in vivo fluorescence imaging of photon‐biotissue interactions in different windows

Herein, we succinctly focus on the recent progress of the different organic fluorescent nanomaterials with NIR‐IIb emission, their unique optical properties, and their application in deep learning in animal models in the NIR‐IIb window (Figure 1A). Lastly, perspectives on the latest challenges and potential future research based on NIR‐IIb fluorescent nanoprobes will also be presented.

## 2 DEEP IMAGING WITH SECOND NEAR‐INFRARED SUB‐WINDOW (NIR‐IIB)

In the last three decades, the search for fluorescent probes with long‐wavelength has spearheaded the discovery of better organic probes such as conjugated polymers, cyanine dyes, and small molecules fluorophores which can be utilized for NIR‐II imaging. The application of NIR‐II imaging has tremendously contributed to the advancement of fundamental research and biomedical field.^[^ ^42^, ^43^ ^]^ Nonetheless, deep imaging with organic fluorescent nanoprobes in the NIR‐II region is unsatisfactory and quite challenging due to insufficient long wavelength, poor optical properties (low stability, and fluorescence quantum yield), or difficult synthesis of organic fluorescent probes.^[^ ^44^ ^]^ Nevertheless, their application for in vivo imaging is more attractive as compared to their inorganic counterparts such as carbon nanotubes and quantum dots. Organic fluorescent nanoprobes are more prone to eradication from the body without a hitch, which makes them have less long‐term toxicity and are more suitable for translation to theranostics application.^[^ ^45^ ^]^

**FIGURE 2:**
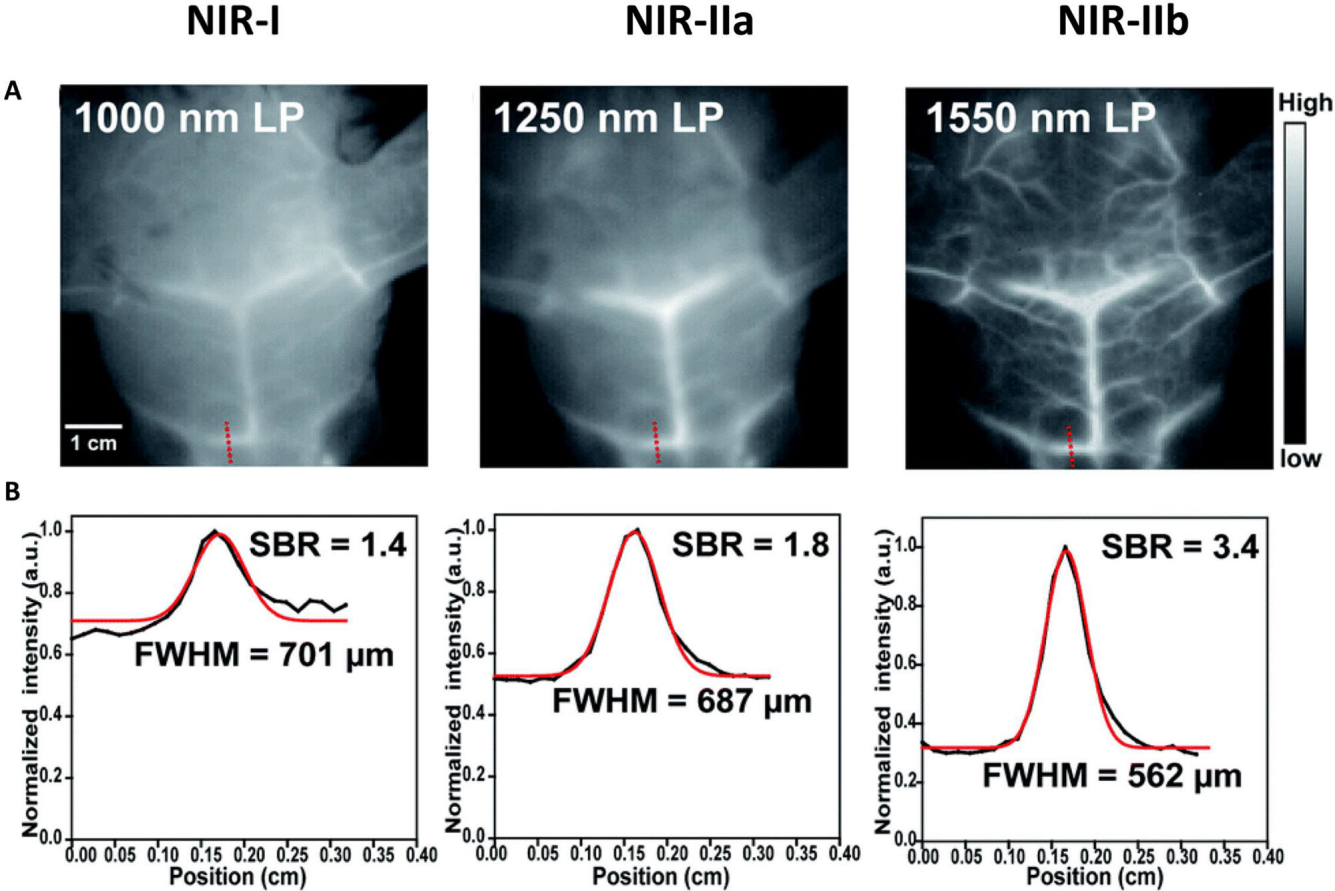
(A) Fluorescence images of mice cerebrovascular (*n* = 3) without craniotomy in different NIR windows. (B) The corresponding signal‐background ratio analysis. Reproduced under the terms of the Creative Commons Attribution 3.0 Unported License with permission.^[^ ^47^ ^]^ Copyright 2020, The Royal Society of Chemistry

Light penetration in biotissues is wavelength‐dependent, therefore, using longer wavelength in the near‐infrared II spectrum translates to reduce photon‐tissue interactions (Figure 1B). In addition, the wavelength is also inversely proportional to the scattering coefficient. Thus, generally, imaging using longer wavelengths enables deep penetration of photons by counteracting photon loss associated with scattering and photon absorption in bio‐tissue. This facilitates the acquisition of top‐notch image quality of deep‐buried events in vivo. Although previously imaging in the NIR‐IIa region outperformed imaging in the UV‐Vis‐NIR‐I window, it still partially suffered from photon dispersion and autofluorescence in biological tissues leading to suboptimal depth penetration and resolution. Meanwhile, imaging beyond the 1500 nm region with NIR‐IIb fluorescent nanoprobes has afforded optimal depth penetration and high spatiotemporal image resolution owing to the longer wavelength that causes suppression of biotissues autofluorescence and light attenuation caused by light scattering and absorption.^[^ ^46^ ^]^ This has permitted the acquisition of unprecedented real‐time high‐quality images (Figure 2A,B).^[^ ^47^ ^]^ Therefore, the NIR‐IIb window is considered to be the best optical biologic window for deep imaging compared to previous windows due to its higher penetration depth and higher resolution (Table 1).^[^ ^48^ ^]^ Furthermore, imaging beyond 1800 nm is impeded by high light attenuation because of increased vibrational overtone caused by liquid water^[^ ^42^ ^]^ Consequently, the utilization of the NIR‐IIb window has made remarkable progress in the acquisition of better image quality in numerous bioimaging applications such as vasculature imaging, in vivo blood circulation tracing, and brain vasculature imaging.

**TABLE 1:** Penetration depth–resolution performance of fluorescence imaging at different windows

**Aspect**		**Visible**	**NIR‐I**	**NIR‐IIa**	**NIR‐IIb**
Penetration depth	Ex vivo	∼2 mm	4–7 mm	∼10 mm	11–20 mm
	In vivo	∼0.1 mm	1–3 mm	∼4 mm	∼3–5 mm
Depth‐to‐resolution ratio	In vivo	∼5	∼15	∼400	∼700

*Source*: Reproduced with permission.^[^ ^48^ ^]^ Copyright 2020, American Chemical Society.

### 2.1 Deep imaging using NIR‐IIb organic nanoparticles‐based fluorescent probes in small animals

Organic fluorescent fluorophores in the second near‐infrared sub‐channel can be classified mainly into two groups. The polymethine (cyanine) and small molecules with donor‐acceptor‐donor (D‐A‐D) structure fluorophores.^[^ ^49^ ^]^ Their fluorescence is governed by their energy bandgap which is closely associated with their conjugated ℼ systems, hence a grasp of the structure‐photophysical property relationship is fundamental in developing superior organic fluorescent nanoprobes.^[^ ^50^, ^51^ ^]^ Therefore, researchers have endeavored to fine‐tune their functionality and photophysical properties via structural engineering and synthetic routes to extrapolate the spectrum range of NIR‐I (700–900 nm) or NIR‐IIa (1000–1300 nm) into NIR‐IIb (1500–1700 nm).^[^ ^52^, ^53^ ^]^ In this review, we focus chiefly on recently reported organic fluorescent nanoprobes that facilitate NIR‐IIb imaging.^[^ ^42^, ^54^, ^55^ ^]^

### 2.2 Polymethine dyes

The polymethine dyes (PMD) are among the brighter organic molecules due to high molar extinction coefficients and narrow energy bandgap and can be bathochromically switched into the NIR‐I channel.^[^ ^56^, ^57^ ^]^ Extension of the polymethine chain or heterocycle substitution strategies is common in red‐shifting their emission resulting in fluorescent probes such as IR26, IR1061, and IR‐12N3 with emission extending into the NIR‐II channel.^[^ ^58^ ^]^ However, from previous studies, it has been substantiated that red‐shifting via extending the polymethine chain can have a negative impact on fluorescence quantum yield and photostability. Hence, red‐shifting of polymethine dyes into NIR‐II via heterocycle substitutions or addition of electron‐donating constituents is more preferred for the development of superior bathochromic‐shifted probes such as Flav7 and FD‐1080.^[^ ^59^, ^60^ ^]^ Thus, these dyes can tail emit in the NIR‐II region exhibiting high extinction coefficients, high quantum yield (QY), and excellent optical stabilities (Table 2). Recently, studies by Carr's group demonstrated that polymethine (cyanine) dye such as ICG, which was traditionally known to emit in the NIR‐I window with a tail emission in the NIR‐II could emit beyond 1500 nm using powerful advanced SWIR cameras such as the indium gallium arsenide (InGaAs).^[^ ^61^, ^62^ ^]^ Thus, repurposing of the NIR‐I dye (ICG) highlighted the importance of fluorophores' optical characterization using superior InGaAs CCD detectors to extend their biomedical application.^[^ ^63^ ^]^ Additionally, the ICG brightness could be enhanced due to improved quantum yield and peak absorption cross‐section in vivo. So far NIR dye ICG has been utilized in the first‐ever tumor‐guided surgery in humans using optical fluorescence imaging.^[^ ^64^ ^]^ But still, the polymethine dyes are not bright enough beyond 1500 nm. Thus, more strategies are needed to construct space‐age polymethine dyes with improved quantum yield and light absorption coefficient.

**TABLE 2:** A brief summary of organic fluorophores with NIR‐IIb emission

**Fluorophore**	**Hydrophilic modification**	**Abs/Em (nm)**	**Quantum yield** **^a^**	**Bioimaging application**	**Ref**.
ICG	Lipophilic	780–820/1500	9.3% (NIR‐I, in serum)	Lymphatic, cardiovascular, intestinal tract tracking, tumor, and whole‐body imaging	[61]
FD‐1080 J‐Aggregates	DMPC	1360–1370/1500	0.0545%	Vascular imaging and hypertension monitoring	[65]
HQL2	DSPE‐mPEG_5k_	710–1050/1500	0.002%	Tumor and vessels imaging	[77]
HL3	DPPE‐_5K_PEG	750–1050/1550	0.005%	Cerebrovascular, lymph node and whole‐body imaging	[47]
2TT‐Oc26b	DSPE‐mPEG_2k_	700–1030/1600	0.012%	Cerebrovascular, intestinal tract tracking, and whole‐body imaging	[81]

^a^ Fluorescence quantum yields of organic fluorophores in the NIR‐IIb region as standardized based on IR26 = 0.05% (in 1,2‐dichloroethane).

In other efforts to improve deep imaging using polymethine‐based probes, Sun's team developed novel NIR‐II FD‐1080 J‐Aggregates that were self‐assembled from a mixture of highly order FD‐1080 (small molecule dye) and a lipid bilayer 1,2‐dimyristoyl‐sn‐glycerol‐3‐phosphocholine (DMPC). This resulted in a superior probe with bathochromically shifted (∼300 nm) absorption and emission of 1360 and 1370 nm, respectively. In comparison to their monomers, the J‐aggregates displayed small stokes shifts of 10 nm, shortened fluorescence lifetime, and negative circular dichroism signals. In addition, they exhibited high absorption coefficients of ∼0.5 × 10^5^ M^−1^ cm^−1^, commendable fluorescence quantum yield of 0.0545%, high hydrophilicity, and better optical stability when compared with their monomers. Thus, the usage of FD‐1080 J‐aggregates enabled deep imaging of the hind limb and brain vasculature beyond 1500 nm with low background interference and high spatiotemporal resolution. Different imaging Windows were used to compare the imaging effects of FD‐1080 J‐aggregates in vivo. Cerebrovascular imaging in the NIR‐IIb displayed a higher signal background of 1.2‐fold and 3.3‐fold increase as compared with imaging in the NIR‐IIa and NIR‐II, respectively. At the same time, the spatial resolution of NIR‐IIb (462 μm) imaging is also higher than that of NIR‐IIa (482 μm) and NIR‐II (502 μm). In imaging of the hindlimb vascular system, similar results were obtained demonstrating superior performance in imaging beyond 1500 nm in the NIR‐IIb window. Based on imaging the carotid artery and monitoring its width change beyond 1500 nm, the efficacy of Isoket (hypotensor) was also assessed in hypertensive rats.^[^ ^65^ ^]^

### 2.3 Small molecules dye with D‐A‐D structure

Over the years, small‐molecule dye with donor‐acceptor‐donor structure pool has expanded exponentially due to their tunable fluorescence spectra, facile functionalization, and high quantum efficiency.^[^ ^66^ ^]^ They include benzobisthiadiazole (BBT), dipyrromethene boron difluoride (BODIPY), benzo[c][1,2,5] thiadiazole (BSBT), squaraine, selenadiazolo[3,4‐*f*] and diketopyrrolopyrrole (DPP)‐base fluorophores with NIR‐II emission.^[^ ^67^ ^]^ The synthesis of small molecules with NIR‐IIb wavelength that can facilitate deep image acquisition with miraculous clarity depends on the optimization of their photoluminescence fluorescence quantum yield (PLQY), brightness, and optical stability (Table 2).^[^ ^44^ ^]^ In relation to the energy gap theory, red‐shifting is at the expense of photoluminescence fluorescence quantum yield. Therefore, the construction of brighter small molecules with D‐A‐D scaffolds that can emit in the NIR‐IIb window requires a deliberate effort of balancing quantum yield and absorption coefficients conundrum. Thus, knowledge and exploitation of the structure‐fluorescence relationship are also crucial in the red‐shifting of small molecule dyes with the Donor–Acceptor–Donor structure. Just like in polymethine dyes, bathochromic shifting via the extension of π‐conjugation in small molecules with a D‐A‐D structure has been reported to also be detrimental. The expansion of π‐π conjugated networks leads to aggregation and quenching, which leads to low photoluminescence quantum yields due to strong intermolecular π‐π stacking promoting non‐radiative route.^[^ ^68^ ^]^ To surmount this, various structural engineering strategies have been applied at a molecular and morphological level such as lodging the molecules with electron donors/acceptors (D/A) substituents which can increase the HOMO and decrease the LUMO levels, respectively. The fabricated molecules have a low energy bandgap that facilitates their red‐shifting as exemplified by the development of NIR‐IIb fluorescent probes, for example, the BBTD‐based fluorophore CH1055.^[^ ^69^ ^]^ But still, most of these fluorescent probes display unsatisfactory low photoluminescence fluorescence quantum yield in aqueous solutions, hence limiting their biological application. To salvage this, other molecular engineering techniques over the years have focused on preventing fluorophores from aggregating, such as introducing shielding units (S) to synthesize S‐D‐A‐D‐S molecules to improve their water solubility and biological applications, but at the expense of absorption coefficients, causing low brightness and interfering with deep imaging.^[^ ^70^, ^71^ ^]^ In 2001, a group led by Tang discovered AIEgens that displayed increased fluorescence upon aggregation due to intramolecular rotation restrictions that permit the radiative channel while closing up the non‐radiative route when they aggregate.^[^ ^72^, ^73^, ^74^ ^]^ AIEgens chromophores exhibited high photoluminescence fluorescence quantum yield beyond the traditional 2%. Thus NIR‐II fluorophores with AIE properties such as BPN‐BBTD, and TB1 are inherently brighter, with high quantum yield and high optical stability.^[^ ^75^, ^76^ ^]^

To expand their repertoire into NIR‐IIb, Li's group exploited the use of NIR‐II fluorophores (Q4) with twisted D‐A architecture and AIE characteristics. They fabricated two NIR‐II fluorophores HQL1 and HQL2. To their surprise upon optical characterization HQL2 displayed high brightness and high fluorescence quantum yield. HQL2 probes exhibited a maximum wavelength of ∼1050 nm with a tail emission of up to 1600 nm. To extend its bioapplication, HQL2 molecules were encapsulated with an amphiphilic molecule DSPE‐PEG5K endowing them with excellent biocompatibility and optical stability. Thereafter, HQL2 dots were applied in dynamic imaging of blood vessels, affording a few millimeters of depth penetration and high clarity in the NIR‐IIb window. The SBR of HQL2 dots was 2.5 times and 1.4 times higher than that of NIR‐II and NIR‐IIb regions when visualized in the NIR‐IIa window. Also, a higher spatial resolution was recorded in the NIR‐IIa (0.345 mm) as compared with NIR‐II (2.13 mm) and NIR‐IIb (0.529 mm). Moreover, upon monitoring of tumor vascular system using the varied channel in the NIR‐II region, the HQL2 dots could only enable delineation of microscopic tumor vessels in mice in the NIR‐IIa (1320 nm) and NIR‐IIb (1550 nm) windows with a high resolution of 0.293 and 0.124 mm, respectively.^[^ ^77^ ^]^ Hence, HQL2 dots have clinical applicability in cancer detection, image‐guided surgery, and treatment assessment.

**FIGURE 3:**
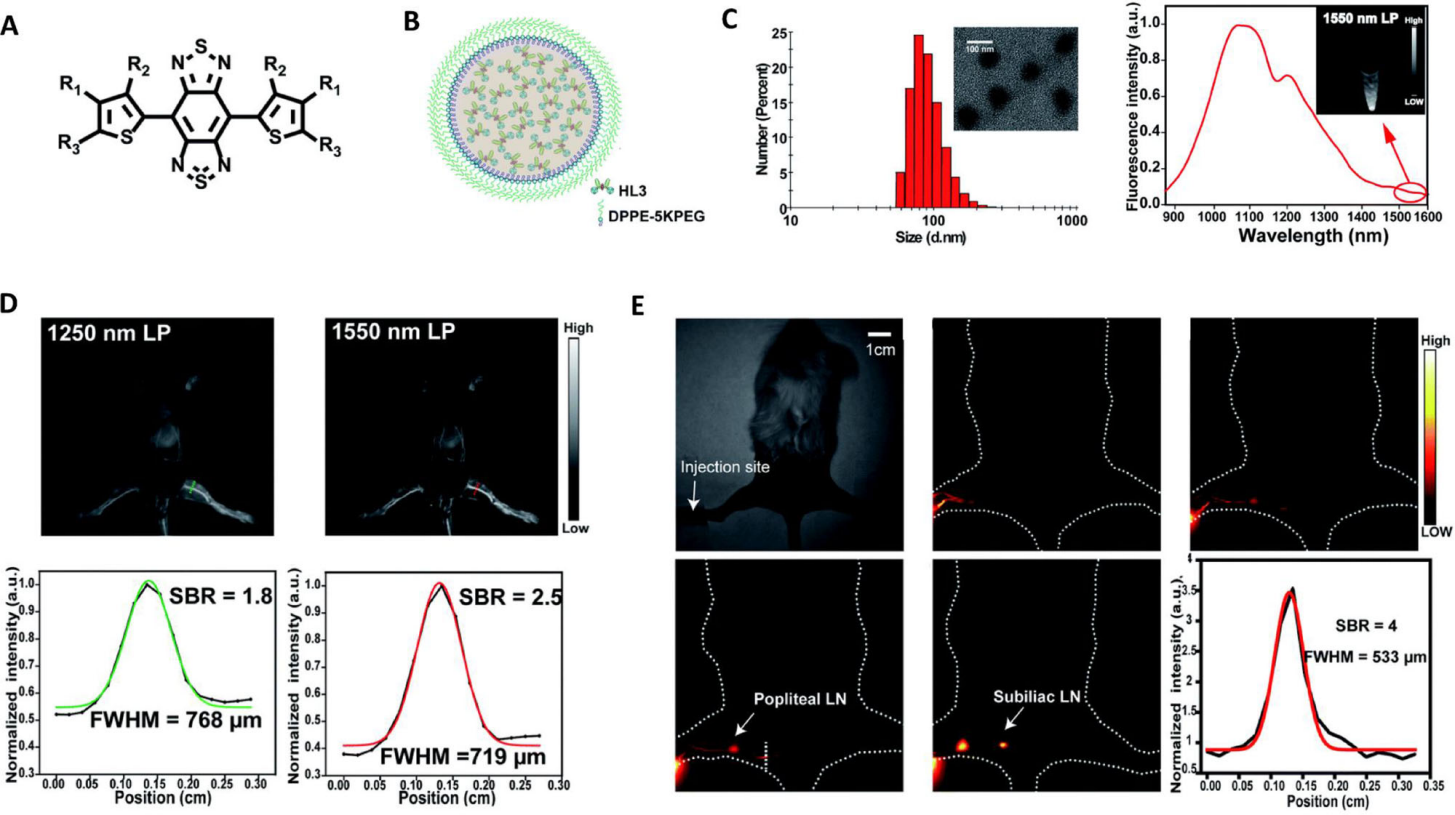
(A) Chemical structure of HL3 molecules. (B) Schematic diagram of HL3 dots. (C) Characterization of HL3 dots using dynamic light scattering, transmission electron microscope (inserted image), and fluorescence spectrum. (D) Fluorescence imaging in the second near‐infrared window of whole mouse blood vessels after tail intravenous injection of HL3 dots at different wavelengths with the corresponding signal‐background ratio analysis, respectively. (E) Dynamic NIR‐IIb imaging of lymphatic drainage in KM normal mice for 3 h after injection of HL3 dots. Reproduced under the terms of the Creative Commons Attribution 3.0 Unported License with permission.^[^ ^47^ ^]^ Copyright 2020, The Royal Society of Chemistry

To further improve imaging in the NIR‐IIb, Li's group developed highly distorted fluorophores HL1‐HL3, based on their previously reported NIR‐II probes Q4 and H1, with a dihedral angle between the acceptor molecule BBTD and donor molecule 3,4‐bis(hexyloxy)thiopene of ∼45°.^[^ ^78^, ^79^ ^]^Among the fluorophore, HL3 exhibited a maximum absorption wavelength of 750 nm and maximum emission at 1050 nm extending up to 1600 nm. Upon encapsulation with amphiphilic DSPE‐PEG5K, the resulting HL3 dots had a quantum yield of ∼0.05% and an absorption coefficient of 9.3 × 10^3^ L mol^−1^ cm^−1^ in an aqueous solution in the NIR‐IIb window (Figure 3A). Furthermore, HL3 dots displayed excellent stability and biocompatibility as long circulation half‐life was recorded. Imaging of the mice blood vessels in the NIR‐IIb (1550 nm) region using HL3 dots exhibited a much higher SBR of 2.5 as compared to imaging in the NIR‐II region using 1000 nm (1.4) and 1250 nm (1.8). Also, a higher spatial resolution at the varied window was recorded with NIR‐IIb 1550 nm (719 μm), NIR‐II 1250 nm (768 μm) NIR‐II 1000 nm (777 μm) (Figure 3D). In imaging, the cerebral vasculature system, a higher SBR (2.4‐fold increase) was recorded in the NIR‐IIb window than that of imaging in the NIR‐II window. Furthermore, a higher spatial resolution was also documented at NIR‐IIb 1550 nm (562 μm), NIR‐II 1250 nm (687 μm), and NIR‐II 1000 nm (701 μm) (Figure 3E). Thus, HL3 dots can not only achieve vascular imaging of the whole body or brain but also achieve high‐resolution imaging of small blood vessels. Hence ,displaying its applicability in investigating biological processes beyond the NIR‐IIb window. Due to the high quantum yield of HL3 dots in the NIR‐IIb window as compared with HQL2 dots.^[^ ^47^ ^]^ HL3 dots were able to facilitate continuous monitoring of the lymph node system with minimum background interference. Thus, proving that NIR‐IIb imaging of HL3 dots has the potential to facilitate prognosis and detection of tumor metastasis at both preclinical and clinical levels.

**FIGURE 4:**
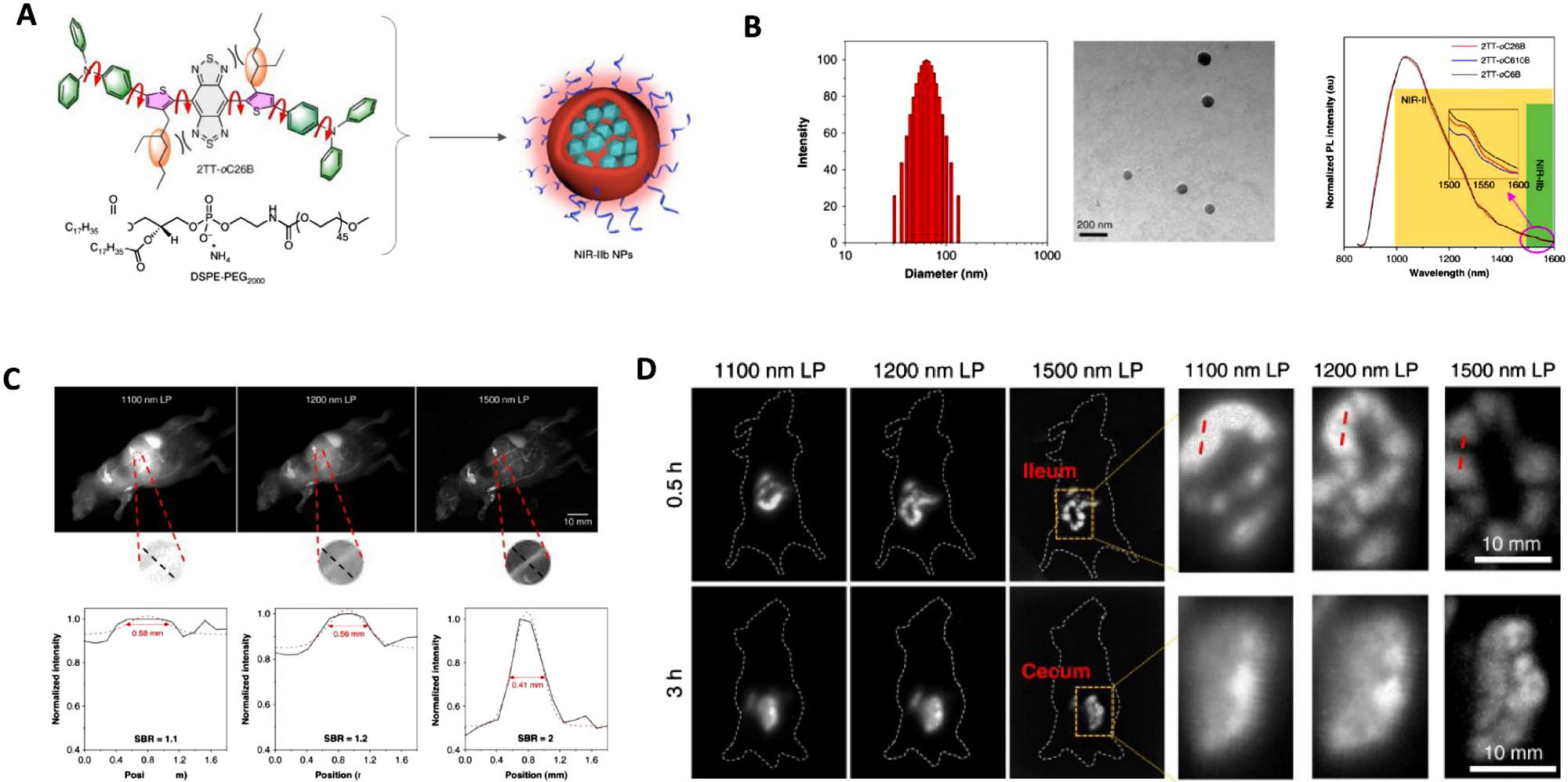
(A) Schematic diagram of encapsulation of 2TT‐oC26B into 2TT‐oC26B nanoparticles. (B) Characterization of 2TT‐oC26B nanoparticles using dynamic light scattering, Transmission electron microscope, and photoluminescence spectra. (C) Whole‐body imaging of living mice near the liver with 2TT‐oC26B NPs in the second window under different long‐pass filters and with the corresponding signal‐background ratio analysis. (D) Near‐infrared‐II fluorescence imaging of the intestinal tract with their corresponding enlarged area. Reproduced under the terms of the Creative Commons Attribution 4.0 International License with permission.^[^ ^81^ ^]^ Copyright 2020, Springer Nature

Even though through twisted intramolecular charge transfer (TICT) manipulation, fluorophores that can tail emit in the NIR‐IIb region are attainable, they are at the expense of fluorescence intensity.^[^ ^80^ ^]^ To overcome this barrier, Tang and his colleges demonstrated that superior organic fluorescent probes would be developed by using TICT and aggregation‐induced luminescence illuminators. Thus, through an elaborate pathway at a molecular (TICT) and morphological (aggregate) level, three distorted NIR‐II fluorophores with AIE characteristics were developed. The intermolecular interactions were lessened to enhance AIE characteristics while restricting close packing leading to the opening up of the radiative decay pathway. As a result, red‐shifted NIR‐II organic fluorescent probes with maximum photoluminescence quantum yield and brightness were constructed. Among the newly developed probes, 2TT‐oC26B fluorescent nanoprobe was more twisted with a higher dihedral angle of ∼48° between BBTD and thiopene (Figure 4A). Additionally, it exhibited a long wavelength that was tailed to 1600 nm in the NIR‐IIb region with a photoluminescence quantum yield (PQY) of 0.12%. In vivo imaging using the 2TT‐oC26B NPs, high‐quality photos images of blood vessels were recorded. Compared with other channels, the NIR‐IIb channel displayed high resolution in imaging blood vessels, indicating its applicability in the utilization of assessing the circulatory system and disease detection (Figure 4C). In addition, it enabled deep visualization of vascular systems adjacent to the liver which is difficult using the traditional NIR‐II channel (Figure 4D). The 2TT‐oC26B NPs also exhibited a higher resolution of ∼71.6 μm in imaging the cerebral vessels which is similar to imaging using inorganic probes. Furthermore, delineation of fine intestinal structure with high resolution in real‐time was possible using NIR‐IIb channel at depth of ∼5 and ∼8 mm, respectively. 2TT‐oC26B NPs were excreted 24 h post gavaging, further indicating their capabilities in deep learning of intestinal diseases in clinic studies.^[^ ^81^ ^]^

## 3 CONCLUSION AND PERSPECTIVES

In the last decade, fluorescence imaging in the second region has made tremendous contributions in propelling deep learning of biological proceedings in living organisms. This has led to the emergency of NIR‐IIb fluorescent probes with longer wavelength emission which results in reduced photon‐biotissues interactions, causing less light absorption, scattering, and tissue autofluorescence. Thus, NIR‐IIb imaging enables to obtain images with much deeper penetration, higher resolution, and better contrast. This state‐of‐art imaging technology displays great potential for future biological imaging. However, it's still at the infancy stage of development. Some issues need to be tackled before they can be widely applied.

First, one of the core challenges revolves around the shortage of fluorescent probes with an inherent longer wavelength. The emission wavelength of organic fluorescent probes depends on their chromophore cores structure. Currently, most polymethine‐based and D‐A‐D‐based probe chromophores are around 1000 nm. Thus, there is a call to further exploit other organic material‐based fluorescent probes to expand the NIR‐IIb probe library.^[^ ^67^ ^]^ Secondly, it is noteworthy that, the current NIR‐IIb fluorescent probes are of unsatisfactory optical characteristics. To optimize their optical properties such as brightness in polar solvents is a huge hurdle. The bottleneck of quantum yield and absorption coefficient should be addressed by improving the organic probes in various areas. For polymethine dyes, extending their chain to lengthen their emission often results in solvatochromic quenching in an aqueous solution. Precisely designing of ℼ‐systems charge, branching the chain, heteroatoms substitution, or strengthening electron‐donating constituents would greatly contribute to their translation into longer wavelength with maximum quantum yield and brightness.^[^ ^59^, ^82^ ^]^ For D‐A‐D‐based dyes, the addition of electron donors and acceptors ℼ spacers should be exploited in red‐shifting their wavelength. Rational molecular engineering strategies such as the addition of shielding units S‐D‐A‐D‐S should be endorsed to enhance their quantum yields and absorption coefficients.^[^ ^70^ ^]^ Another strategy may be to combine twisted intramolecular charge transfer with aggregation‐induced emission for constructing novel fluorescent probes.^[^ ^81^ ^]^ These will aid in developing novel NIR‐IIb nanoprobes with long‐wavelength far beyond 1500 nm, high quantum yield, and excellent brightness. Thirdly, the lack of functional constituents to enable facile functionalization is another obstacle in their utilization for NIR‐IIb probes. Thus, novel strategies should also be developed to facilitate easy functionalization of the probes which would improve their targeting ability.^[^ ^83^ ^]^ Lastly, biocompatibility is also an important issue for the application of NIR‐IIb nanoprobes. For example, surface coating with amphipathic polymers such as DSPE‐PEG and PS‐PEG or serum such as FBS or HSA has shown potential in improving their biocompatibility as well as quantum yield. Furthermore, size optimization to facilitate renal excretion should be put into consideration to improve their pharmacokinetics when designing new probes.^[^ ^34^ ^]^

New functional NIR‐IIb probes could also be developed to promote the performance of near‐infrared II sub‐window imaging in living organisms. For instance, NIR‐IIb probes usually have only the switch on mode. To optimize their imaging precision, activatable nanoprobes can be exploited to visualize physiological processes with a high signal/background ratio.^[^ ^84^, ^85^ ^]^ To improve their capabilities in multispectral imaging, NIR‐IIb probes with large stoke‐shift and different spectrums should be designed to avoid crosstalk. Furthermore, exploiting multifunctional organic fluorescent nanoprobes that combine NIR‐IIb imaging and therapeutic properties is also a good strategy, which will provide new in vivo theranostics platforms.^[^ ^86^ ^]^ Additionally, NIR‐IIb imaging integration with other clinical techniques needs to be exploited. Incorporating clinical procedures such as MRI and CT with NIR‐IIb imaging would be a great approach to simultaneously obtain anatomical structure and functional information in living organisms. This would greatly benefit disease diagnosis and treatment.^[^ ^87^ ^]^

NIR‐IIb organic fluorescent nanoprobes hold great potential in various areas of research and clinical application. One application that would greatly benefit from NIR‐IIb imaging advancements is tumor diagnosis and treatment. For precise tumor diagnosis and effective treatment, tumor development and metastasis processes need to be comprehensively elucidated. This is not yet possible with the current probes. The incorporation of NIR‐IIb imaging with multispectral imaging approaches would provide a good platform for deciphering tumor development and metastasis.^[^ ^88^ ^]^ Image‐guided tumor therapy is another area that the NIR‐IIb imaging has immense potential in the future. Its superiority in deep imaging and commendable spatiotemporal resolution would greatly aid to improve precision in tumor resection.^[^ ^89^ ^]^ Still, the advancement of NIR‐IIb nanoprobes with improved tumor selectivity would facilitate tumor treatment using photodynamic or photothermal therapies.^[^ ^90^, ^91^ ^]^ This will provide more effective and safer approaches to treat tumors. NIR‐IIb imaging also has immense potential in basic research areas. For example, till now, it's not yet feasible to non‐invasively elucidate the embryonic process and organ development process in living organisms. The employment of NIR‐IIb probes with the incorporation of multispectral imaging approaches would aid in obtaining the intriguing dynamic processes of development. The utilization of NIR‐IIb imaging can also help to elucidate the dynamic differentiation and fate of stem cells.^[^ ^92^ ^]^ In the neuroscience field, NIR‐IIb imaging also exhibits promising potential. For instance, to detect ions and neurotransmitters (Ca^2+^, K^+^, and dopamine) that influence neural activity, activatable NIR‐IIb probes will be powerful tools that switch on upon sensing the ions and neurotransmitters. In addition, NIR‐IIb imaging would facilitate the decipherment of the underlying mechanisms involved in neurological diseases.^[^ ^40^ ^]^ It will broaden our understanding and promote the development of cutting‐edge therapies.

All in all, NIR‐IIb nanoprobes have a great potential in deep imaging by overcoming biological tissue impediments, which can facilitate new explorations in basic research and hasten their theranostics applications in clinics.

## CONFLICT OF INTEREST

Zongqiang Cui is a member of the *Exploration* editorial board. The authors declare no conflict of interest.
